# Exploring Muscle Health Deterioration and Its Determinants Among Community-Dwelling Older Adults

**DOI:** 10.3389/fnut.2022.817044

**Published:** 2022-04-29

**Authors:** Yuan-Ping Chao, Wen-Hui Fang, Wei-Liang Chen, Tao-Chun Peng, Wei-Shiung Yang, Tung-Wei Kao

**Affiliations:** ^1^Division of Family Medicine, Department of Family and Community Medicine, Tri-Service General Hospital, School of Medicine, National Defense Medical Center, Taipei, Taiwan; ^2^Division of Geriatric Medicine, Department of Family and Community Medicine, Tri-Service General Hospital, School of Medicine, National Defense Medical Center, Taipei, Taiwan; ^3^Graduate Institute of Medical Sciences, National Defense Medical Center, Taipei, Taiwan; ^4^Graduate Institute of Clinical Medicine, College of Medicine, National Taiwan University, Taipei, Taiwan; ^5^Department of Internal Medicine, National Taiwan University Hospital, Taipei, Taiwan; ^6^Center for Obesity, Life Style and Metabolic Surgery, National Taiwan University Hospital, Taipei, Taiwan

**Keywords:** sarcopenia, transition, muscle function, fat-to-muscle ratio, dynapenia

## Abstract

**Background:**

Age-related muscle mass and function decline are critical issues that have gained attention in clinical practice and research. Nevertheless, little is known regarding the time course of muscle health progression, and its determinants during this transition should be estimated.

**Methods:**

We enrolled community-dwelling adults aged ≥65 years during their regular health checkup. The participants’ body composition and muscle function were measured annually from 2015 to 2021. Presarcopenia was characterized by the loss of muscle mass only; dynapenia was defined as low muscle function without changes in muscle mass; and sarcopenia was indicated as a decline in both muscle mass and muscle function. We observed the natural course of muscle health progression during aging. The relationship between muscle health decline and different determinants among old adults was examined.

**Results:**

Among 568 participants, there was 18.49%, 3.52%, and 1.06% of healthy individuals transited to dynapenia, presarcopenia, and sarcopenia, respectively. Significant positive correlations between age, fat-to-muscle ratio (FMR) and the dynapenia transition were existed [hazard ratio (HR) = 1.08 and HR = 1.73, all *p* < 0.05]. Serum albumin level had negative correlation with the dynapenia transition risk (HR = 0.30, *p* = 0.004). Participants with these three risk factors had the highest HR of dynapenia transition compared to those without (HR = 8.67, *p* = 0.001). A dose-response effect existed between risk factors numbers and the risk of dynapenia transition (*p* for trend < 0.001). This positive association and dose-response relationship remains after multiple covariates adjustment (HR = 7.74, *p* = 0.002, *p* for trend < 0.001). Participants with two or more than two risk factors had a higher risk of dynapenia transition than those with low risk factors (*p* = 0.0027), and the HR was 1.96 after multiple covariate adjustment (*p* = 0.029).

**Conclusion:**

Healthy community-dwelling old adults tended to transit to dynapenia during muscle health deterioration. Individuals with older age, higher FMR, lower albumin level had a higher risk of dynapenia transition; and a positive dose-response effect existed among this population as well.

## Introduction

The distressing physical challenges of advancing age include progressively decline in muscle mass and muscle function with concomitant increase in body fat, which result in adverse clinical events such as falls, fractures, functional disability, and mortality (1–3). The term sarcopenia was originally meant to represent the loss of muscle mass during aging process (4). To facilitate the precise identification of deteriorated muscle health, more definitions have been proposed. According to the consensus in 2010 European Working Group on Sarcopenia in Older People (EWGSOP), presarcopenia was defined as decreased muscle mass with intact muscle strength and physical performance, and sarcopenia was described as low muscle mass with decline in muscle strength and/or physical performance (5). In 2014, Asian Working Group for Sarcopenia (AWGS) also declared Asian consensus regarding clinical operational guidelines on sarcopenia assessment with specific cutoff values for three muscle health parameters (6). Both of muscle quantity and muscle quality are equally important in muscle health aging since then.

Nevertheless, there is plenty of evidence supporting disassociated causality between changes in muscle mass and muscle strength during aging; furthermore, it revealed that the contribution of muscle atrophy to the decline in muscle strength is relatively sparse (7, 8). In reality, Clark et al. has proposed the term “dynapenia” in 2008 to define the loss of muscle strength and muscle power during aging (9). Hence, the occurrence of muscle mass loss and muscle function decline and their impacts during aging process attracted more attention in clinical research and practice.

In addition to the decline in muscle mass and muscle strength, increased fat composition during aging was also correlated with poor muscle health, and this issue has recently acquired more attentions. Based on the 1999–2002 National Health and Nutrition Examination Survey (NHANES), Danielle R Bouchard and colleagues examined the relationship among muscle strength loss, obesity, and physical function. They suggested that dynapenic obesity was associated with poorer physical performance than obesity or dynapenia alone (10). High fat mass and low muscle strength were also correlated with impaired physical performance (11–13). Therefore, body fat composition should be taken into consideration while assessing muscle health deterioration in geriatric care.

Nonetheless, little is known about the sequential order of occurrence in muscle health changes along with aging, and the major determinants associated with this process are rarely to be explored. To investigate the time course of muscle health decline, we aimed to observe changes in muscle mass, muscle function and the risk factors including different obesity indicators among Taiwanese community-dwelling older adults. We hypothesized that muscle function decline supposed be more prevalent and earlier than muscle mass loss, and the higher obesity indicators determined the muscle health deterioration during aging process.

## Materials and Methods

### Study Design and Participants

Community-dwelling old adults aged 65 years and older were enrolled in this study. They had undergone a general health checkup at the health check center in Tri-Service General Hospital (TSGH) in Taiwan from 2015 to 2021. We traced the participants and observed the parameters of skeletal muscle mass, muscle strength and physical performance annually. Participants with initial robust status transited to dynapenia, presarcopenia or sarcopenia were treated as the censoring indicator. Data were retrieved from a comprehensive questionnaire, including physical function and biological indicators. Participants with the following conditions were excluded: chest or joint pain during exercise; cognitive impairment; history of heart failure; current history of cancer under treatment; history of renal failure under regular hemodialysis; and exercise not recommended by physicians. Based on the revised Helsinki Declaration, the Institutional Review Board (IRB) of TSGH approved this study. Informed consent was obtained from all the participants.

### Measurement of Muscle Strength, Physical Performance and Muscle Mass

The handgrip strength of the dominant hand was assessed with a handheld dynamometer. The time required by the participants to walk 6 m at the usual pace was recorded by a handheld chronograph, and gait speed was calculated. Eight-electrode bioelectrical impedance analysis (BIA, InBody720, Biospace, Seoul, South Korea) (14) was applied to measure appendicular skeletal muscle mass (ASM), and height-adjusted skeletal muscle mass (ASM/m^2^) was calculated to define the skeletal muscle mass index (SMI).

### Measurement of Obesity Profiles

Clinical measurements are all followed by standard recommended procedures. Body mass index (BMI) was defined as body weight in kilograms divided by body height in square meters (kg/m^2^). Waist circumference (WC) was measured at the mid-level between the lowest costal margin and the highest border of iliac crest when the participant in the standing position. Body fat mass and body fat percentage were measured by BIA (14). Body fat mass divided by body skeletal muscle mass was calculated as the fat-to-muscle ratio (FMR).

### Definitions of Dynapenia, Presarcopenia, and Sarcopenia

The cutoff points of low SMI, low handgrip strength and slow gait speed were determined according to the 2019 AWGS consensus (15). Sarcopenia was defined as low SMI plus low handgrip strength and/or slow gait speed. Presarcopenia was defined as low SMI without a decline in handgrip strength or gait speed. Dynapenia was defined as low handgrip strength and/or slow gait speed without low SMI.

### Covariates Measurement

Age, sex, smoking status, alcohol consumption, and medical history were collected from a self-completed questionnaire and health insurance card. Past history, including hypertension, diabetes mellitus (DM), stroke, coronary artery disease (CAD), chronic obstructive pulmonary disease (COPD), and arthritis, was obtained by self-report of a doctor’s diagnosis. We applied the Chinese version of the International Physical Activity Questionnaire (IPAQ) for physical activity evaluation (16). After the patients fasted for 8 h, blood samples were collected. White blood cell counts, hemoglobin, serum low-density lipoprotein cholesterol (LDL), serum creatinine, and serum albumin levels were analyzed.

### Statistical Analysis

Statistical analyses were performed with SPSS (Version 18.0 for Windows; SPSS Inc.). We calculated the mean ± standard deviation values for each continuous variable and numbers (percentages) for categorical variables. The transition rates from robust to dynapenia, presarcopenia and sarcopenia were calculated. The covariates with significant hazard ratio to increase the risk from robust transition to dynapenia were determined by Cox regression analysis. In addition to the possible confounding factors such as age, sex, chronic diseases, physical activity and nutrition related biomarkers, we especially focused on different fat indices including BMI, WC, fat mass, body fat percentage and FMR to investigate their impact on muscle health deterioration. The cutoff point of the high risk factor with significant hazard ratio to predict dynapenia transition was determined by using receiver operating characteristic (ROC) analysis. Kaplan–Meier analysis was applied to ascertain the relation of high/low risk factor and the cumulative hazard of transition to dynapenia. The effects of cluster risk factors compared to no risk factor on dynapenia transition were analyzed by multiple covariates Cox regression. To determine the effects of high risk factors on the risk prediction of dynapenia transition, the extended model approach with stepwise variable adjustment by Cox proportional hazards regression was conducted as follows: Model 1 = sex, smoking, alcohol consumption; Model 2 = Model 1 + hypertension, DM, stroke, CAD, COPD, arthritis; Model 3 = Model 2 + physical activity, WBC counts, hemoglobin, serum creatinine, LDL

## Results

### Demographics of Participants by Robust Progression to Dynapenia, Presarcopenia, and Sarcopenia

The demographics of the 568 subjects by transition status are presented in Table 1. The mean age was 70.09 ± 5.46 years, and 49.82% (*n* = 283) of the participants were male. During the 6-year follow-up period, 18.49% (*n* = 105), 3.52% (*n* = 20), and 1.06% (*n* = 6) of individuals transited to dynapenia, presarcopenia, and sarcopenia, respectively. The mean time of transition to dynapenia was 63.93 ± 18.97 months, to sarcopenia was 71.46 ± 5.27 months, to presarcopenia was 70.43 ± 8.45months. There were significant differences among the transitional statuses in age, WC, BMI, SMI, fat mass, physical activity, albumin, and history of COPD. Individuals who transited to dynapenia were tended to be older and had a higher obesity profile (BMI, WC, fat mass, body fat percentage, and FMR) as well as a lower albumin level.

**TABLE 1:** Characteristics of participants from robust transition to dynapenia, presarcopenia and sarcopenia.

		Transition status			
	
Initial status Robust (N = 568)	Robust *n* = 437 76.94%	Dynapenia *n* = 105 18.49%	Presarcopenia *n* = 20 3.52%	Sarcopenia *n* = 6 1.06%	
**Continuous variables*^a^***					***p-*** **value**
Age (years)	69.5 ± 5.0	72.3 ± 6.1	72.9 ± 7.1	66.8 ± 6.4	< 0.001
WC(cm)	82.6 ± 9.1	82.9 ± 8.1	76.3 ± 8.9	79.8 ± 5.2	0.014
BMI (kg/m^2^)	25.12 ± 3.08	25.58 ± 2.61	22.78 ± 2.58	23.42 ± 1.54	0.001
SMI (kg/m^2^)	7.15 ± 0.92	7.16 ± 0.91	6.54 ± 0.85	6.66 ± 0.72	0.018
Grip strength (kg)	30.8 ± 8.8	29.1 ± 8.4	28.7 ± 8.4	29.2 ± 9.5	0.259
Gait speed (m/s)	1.32 ± 0.25	1.27 ± 0.21	1.42 ± 0.26	1.31 ± 0.18	0.076
Fat mass (kg)	19.7 ± 6.1	21.0 ± 5.6	16.1 ± 4.8	16.7 ± 4.7	0.004
Body fat (%)	29.9 ± 7.4	31.4 ± 6.9	27.8 ± 6.4	28.0 ± 7.9	0.107
FMR	0.82 ± 0.32	0.89 ± 0.30	0.73 ± 0.24	0.75 ± 0.31	0.123
PA (kcal/week)	10411.0 ± 3586.1	8507.3 ± 2670.8	8419.7 ± 3534.6	9011.0 ± 3598.1	0.002
WBC counts (/ul)	5721 ± 1413	5851 ± 1417	5363 ± 1380	6187 ± 1360	0.497
Hemoglobin (g/dl)	14.0 ± 1.1	13.7 ± 1.3	13.4 ± 0.8	14.6. ± 0.1	0.063
Creatinine (mg/dl)	0.90 ± 0.59	0.94 ± 0.73	0.88 ± 0.22	0.72 ± 0.22	0.867
LDL (mg/dl)	110.5 ± 30.7	104.0 ± 26.1	101.1 ± 27.7	126.0 ± 23.9	0.110
Albumin (mg/dl)	4.34 ± 0.21	4.28 ± 0.25	4.24 ± 0.30	4.38 ± 0.16	0.028
**Categorical variables *^b^***					
Male	225 (51.5)	47 (44.8)	8 (40.0)	3 (50.0)	0.506
Smoking	102 (23.3)	21 (20.0)	2 (10.0)	2 (33.3)	0.441
AC ≥ 1 time/week	43 (9.8)	14 (13.3)	2 (10.0)	1 (16.7)	0.704
Hypertension	146 (33.4)	41 (39.0)	5 (25.0)	1 (16.7)	0.432
DM	41 (9.4)	14 (13.3)	2 (10.0)	1 (16.7)	0.607
Stroke	6 (1.4)	2 (1.9)	0	0	0.906
CAD	19 (4.3)	8 (7.6)	0	0	0.349
COPD	12 (2.7)	6 (5.7)	2 (10.0)	2 (33.3)	0.023
Arthritis	57(13.0)	19 (18.1)	4 (20.0)	2 (33.3)	0.224

*AC, alcohol consumption; BMI, body mass index; CAD, coronary artery disease; COPD, chronic obstructive pulmonary disease; DM, diabetes mellitus; FMR, fat-to-muscle ratio; PA, physical activity; LDL, low-density lipoprotein; SMI, skeletal muscle mass index; WBC, white blood cell; WC, waist circumference.*

*^a^Values in the continuous variables were expressed as mean ± standard deviation.*

*^b^Values in the categorical variables were expressed as number (percent).*

### Association Between Covariates and the Risk of Transition to Dynapenia

Age, fat mass, body fat percentage, and FMR showed significant positive correlations with the risk of transition to dynapenia [Hazard ratio (HR) = 1.08, 95% confidence interval (CI) = 1.05–1.11, *p* < 0.001; HR = 1.04, 95%CI = 1.01–1.07, *p* = 0.021; HR = 1.03, 95%CI = 1.01–1.05, *p* = 0.034; and HR = 1.73, 95%CI = 1.05–2.84, *p* = 0.029, respectively). Serum albumin level had significant negative correlation with the risk of dynapenia transition (HR = 0.30, 95%CI = 0.13–0.67, *p* = 0.004). However, the relationships between BMI and WC and the risk of transition to dynapenia were insignificant (Table 2). Among the obesity parameters, FMR had the highest HR of dynapenia transition, and the optimal cutoff value of FMR for this risk prediction was 0.863 by using ROC analysis. The optimal cutoff point of age to estimate dynapenia transition was 68.5 years old, and the optimal cutoff value of serum albumin was 4.35mg/dl. Participants with an older age, high FMR and low albumin level were defined accordingly for further analysis.

**TABLE 2:** Hazard of covariates in participants transition from robust to dynapenia.

	Hazard ratio (95% confidence interval)	*p*-Value
Age	1.08 (1.05–1.11)	< 0.001
Sex	0.86 (0.58–1.27)	0.464
smoking	0.97 (0.60–1.57)	0.917
AC ≥ 1 time/week	1.47 (0.83–2.58)	0.182
Hypertension	1.34 (0.90–2.00)	0.143
DM	1.45 (0.82–2.55)	0.199
Stroke	1.50 (0.37–6.11)	0.566
CAD	1.85 (0.90–3.82)	0.093
COPD	1.68 (0.73–3.85)	0.214
Arthritis	1.29 (0.78–2.12)	0.318
Body mass index	1.06 (0.99–1.13)	0.058
Waist circumference	1.01 (0.98–1.03)	0.355
Fat mass	1.04 (1.01–1.07)	0.021
Body fat percentage	1.03 (1.01–1.05)	0.034
FMR	1.73 (1.05–2.84)	0.029
White blood cell counts	1.13 (0.98–1.29)	0.086
Hemoglobin	0.89 (0.72–1.09)	0.272
Serum creatinine	1.11 (0.86–1.41)	0.426
LDL	0.99 (0.98–1.00)	0.088
Albumin	0.30 (0.13–0.67)	0.004

*AC, alcohol consumption; CAD, coronary artery disease; COPD, chronic obstructive pulmonary disease; DM, diabetes mellitus; FMR, fat-to-muscle ratio; LDL, low-density lipoprotein.*

### Association Between Cluster of Risk Factors and the Risk of Transition to Dynapenia

Older age, higher FMR and lower albumin level were taken into account as risk factors to predict dynapenia transition. There were 540 participants with complete data of these three parameters enrolled for subgroup analysis. Participants with three risk factors had the highest HR of dynapenia transition compared to the participants with no risk factor (HR = 8.67, 95%CI = 2.50–30.04, *p* = 0.001) (Table 3). A dose-response effect was noted between risk factors numbers and the risk of dynapenia transition (*p* for trend < 0.001). The positive association remains after further adjustment with multiple covariates (HR = 7.74, 95%CI = 2.16–27.58, *p* = 0.002). And the dose-response relationship between numbers of risk factors and the transition risk to dynapenia still unchanged (*p* for trend < 0.001).

**TABLE 3:** Hazard ratio of cluster risks to predict participants transition from robust to dynapenia.

	Hazard ratio (95% confidence interval)	*p*-Value	*p* for trend
**Model 1**			
No risk factor	Reference		<0.001
1 Risk factor	3.22 (0.93–11.08)	0.064	
2 Risk factors	3.72 (1.10–12.56)	0.034	
3 Risk factors	8.67 (2.50–30.04)	0.001	
**Model 2**			
No risk factor	Reference		<0.001
1 Risk factor	2.93 (0.84–10.14)	0.091	
2 Risk factors	3.44 (1.01–11.67)	0.048	
3 Risk factors	7.84 (2.23–27.43)	0.001	
**Model 3**			
No risk factor	Reference		<0.001
1 Risk factor	2.90 (0.82–10.13)	0.096	
2 Risk factors	3.25 (0.92–11.37)	0.065	
3 Risk factors	7.74 (2.16–27.58)	0.002	

*Model 1 = sex, smoking, AC.*

*Model 2 = Model 1 + hypertension, DM, stroke, CAD, COPD, arthritis.*

*Model 3 = Model 2 + PA, WBC counts, Hemoglobin, serum creatinine, LDL. AC, alcohol consumption; CAD, coronary artery disease; COPD, chronic obstructive pulmonary disease; DM, diabetes mellitus; PA, physical activity; LDL, low-density lipoprotein; WBC, white blood cell.*

### Cumulative Risk of Transition to Dynapenia Between High Risk Factor and Low Risk Factor

**FIGURE 1:**
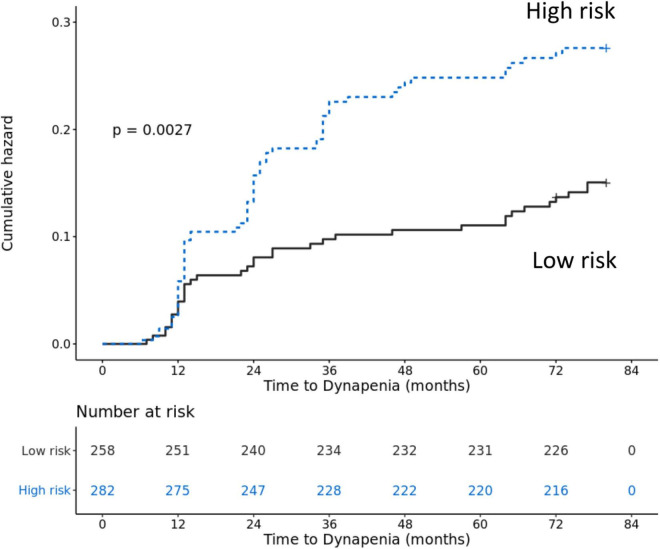
Accumulative risk of transition to dynapenia from robust between participants with high risk factors and low risk factors.

Participants with two or more than two risk factors of dynapenia transition were defined as high risk factors. Figure 1 shows the Kaplan–Meier analysis to estimate the 78 months of cumulative hazard on transition to dynapenia by high and low risk factors. Participants with high risk factors had a higher risk of transition to dynapenia than those with low risk factors (*p* = 0.0027).

### The Risk of Transition to Dynapenia by High Risk Factor

Multiple covariate Cox regression analysis was performed to examine the association of high risk factors and the risk of transition to dynapenia (Table 4). After multiple covariate adjustment, the HR of transition to dynapenia for participants with high risk factors was 1.96 (95%CI = 1.07–3.59, *p* = 0.029).

**TABLE 4:** Hazard ratio of transition from robust to dynapenia in participants with high risk factors.

	Hazard ratio (95% confidence interval)	*p*-Value
Model 1	2.06 (1.17–3.64)	0.012
Model 2	2.01 (1.12–3.57)	0.018
Model 3	1.96 (1.07–3.59)	0.029

*Model 1 = sex, smoking, AC.*

*Model 2 = Model 1 + hypertension, DM, stroke, CAD, COPD, arthritis.*

*Model 3 = Model 2 + PA, WBC counts, Hemoglobin, serum creatinine, LDL. AC, alcohol consumption; CAD, coronary artery disease; COPD, chronic obstructive pulmonary disease; DM, diabetes mellitus; PA, physical activity; LDL, low-density lipoprotein; WBC, white blood cell.*

## Discussion

Among the community-dwelling older people included in this study, 76.94% remained healthy with good muscle mass and muscle function at the 6-year follow-up. Regarding individuals with muscle health progression, 18.49% of old adults transited to dynapenia, followed by presarcopenia (3.52%) and sarcopenia (1.06%). The analyses suggested that participants had more problems in terms of muscle strength decline rather than muscle mass loss during aging process, which was consistent with the findings of some longitudinal studies (9, 17, 18). For example, Visser M and colleagues found that participants had a greater decrease in knee extensor strength than in the mid-thigh muscle cross-sectional area (CSA) in both sexes in a 2.5-year longitudinal study (19). Delmonico et al. observed that there were 9 and 3.2% reductions in the total thigh area among men and women during a 5-year follow-up; nevertheless, the loss of average maximal muscle torque was much greater than the total thigh area, with 16.1% in men and 13.4% in women (18). Clark et al. investigated muscle mass and knee extensor strength among elderly individuals in a 6-year cohort study and showed that participants with different annual rates of muscle strength decline had similar amounts of muscle mass loss (9). According to the study by Murphy et al., gait speed and handgrip strength decline before the loss of appendicular lean mass during the transition from healthy muscle conditions to sarcopenia state (20). Based on a 12-year follow-up cohort study, Frontera et al. found a much greater decrease of 20–30% in knee and elbow isokinetic muscle strength than the loss of 10–15% in mid-thigh muscle CSA among older men (17). To the best of our knowledge, studies applying the cutoff point of low muscle mass, low muscle strength and slow gait speed in light of the EWGSOP or AWGS consensus are scarce. For a more comprehensive survey, we used the latest AWGS consensus to define low muscle function and low muscle mass for observing muscle health deterioration from robust to dynapenia, presarcopenia, and sarcopenia.

In addition to muscle function decline, we found that individuals who transited to dynapenia had a higher obesity profile. This result was in line with that of a cross-sectional study, which suggested that older adults with a higher body fat percentage tend to have a lower handgrip strength and walking speed than those with a lower body fat percentage (21). In addition, declines in handgrip strength and gait speed accelerate while fat mass increases in people aged 70–75 years (22). This result was also supported by our earlier report that handgrip strength and gait speed decreased as FMR and body fat percentage increased in elderly individuals (23). Studies concerning the relationship between other obesity indicators and muscle function showed similar outcomes. A negative correlation was observed between WC and handgrip strength in a Taiwanese cohort study (24). Individuals with obesity at 20–50 years of age (defined by BMI ≥ 30 kg/m^2^) were associated with low handgrip strength (25). The consistent findings across past studies using various methods for obesity and muscle strength measurement are remarkable and lend support for the findings of the present study. The novelty of our research was the application of the latest AWGS cutoff points for low muscle strength and low muscle mass. To the best of our knowledge, this is the first longitudinal study comparing multiple obesity indicators during the natural course of muscle health transition to dynapenia, presarcopenia and sarcopenia.

In our study, the correlations between BMI, WC and the transition to dynapenia were insignificant. Skeletal muscle mass may decrease by up to 40% between 20 and 70 years of age, and fat mass will increase to a peak gradually at the age of 60–70; then, fat mass and fat-free mass will decline simultaneously later in life (26–28). Older individuals tend to have greater amounts of visceral fat than younger subjects despite having the same WC (29). It was also suggested that BMI is an imperfect fat index for older adults in research (30). Using BMI and WC as fat indicators may be masked by body composition changes during aging. These could partially explain the insignificant findings of the correlations between BMI, WC and the transition to dynapenia.

On the other hand, significantly positive correlations existed between fat mass, FMR, body fat percentage and the transition to dynapenia. This result suggested that taking fat components into consideration is important when evaluating muscle strength and gait speed deterioration. Among the fat indicators, FMR was found to be the best predictor for the risk of transition to dynapenia. Lee HS et al. also explored that FMR was negatively correlated with handgrip strength in both sexes, and participants in the highest FMR quartile had lower handgrip strength among 55- to 65-year-old patients undergoing hemodialysis in Korea (31). According to the study by Sternfeld et al., the lean-to-fat ratio was positively associated with walking speed among community-dwelling participants aged 55 years and older in California (32). In their opinion, muscle strength or function could be assessed more precisely by considering body fat, and this concept was in accordance with our results. Similarly, the two abovementioned studies both applied BIA and calculated the ratio of body fat mass to muscle mass. The two studies focused on the correlations of FMR with physical performance, cardiac events, and all-cause mortality. In contrast, our study emphasized the relationship between obesity indicators and the natural course of muscle health transition. Moreover, we applied AWGS cutoff points to define low muscle quantity and low muscle quality. In this context, the FMR, which simultaneously considers muscle mass and fat mass, may provide a more suitable surrogate for estimating muscle health transition to dynapenia. Previous literature also provided a possible mechanism linking fat infiltration into the muscle, muscle function decline, and metabolic disarrangement (33). Intermuscular and intramuscular fat accretion coincides with aging and obesity and may be associated with deleterious health events (18, 34, 35). The infiltrated fat may induce lipotoxicity, which is crucial in the development of peripheral insulin resistance during the aging process (35–37). Muscle contraction leads to energy depletion and stimulates 5′-adenosine monophosphate-activated protein kinase (AMPK) activity to facilitate ATP synthesis (38, 39). Obesity suppresses AMPK activity (40), promoting fast-to-slow muscle fiber transformation, which can affect energy use and contractile function (41, 42). Obesity is also related to increases in pro-inflammatory cytokines, such as interleukin-6 (IL-6) and tumor necrosis factor-alpha (TNF-α) (43). These cytokines are associated with lower muscle mass and strength in the elderly, and the possible mechanism may be muscle protein catabolism and inhibiting muscle protein synthesis (44). In summary, obesity and fatty deposits in the muscle directly induce inflammation, which in turn negatively interferes with muscle strength (43).

In addition to older age and adiposity, we also explored that low albumin level increased the risk of dynapenia transition. Protein synthesis with energy production is necessary for muscle strength generation. Older people with malnutrition will increase the risk of frailty, disability and mortality. Some study results are consistent with our research findings. Schalk et al. explored that lower serum albumin level was cross-sectionally associated with handgrip strength decline at baseline in both genders with age of 65–88 years old. After adjustment with multiple confounders, the substantial negative relationship remained between low serum albumin concentration and handgrip strength over 3- and 6-year follow up (45).

In a prospective cohort study from community-dwelling people aged 55 and older over with 3–5 years follow-up, it revealed that older adults with low concentration of serum albumin will decrease 37% in gait speed and 91% in timed-up-and-go test (46). To sum up, malnutrition represented by low serum albumin level is an optimal surrogate to predict muscle function transition from robust to dynapenia.

In our study, older age, high FMR and low serum albumin level were the three risk factors leading to increase the risk of muscle health transition from robust to dynapenia. A significant dose-response effect existed between cluster factors and the risk of dynapenia transition. In general, older adults with two or more than two of these factors will increase the dynapenia transition risk than those without. This is the first study to composite the possible risk factors in predicting dynapenia transition among older people.

The strength of this study is that we explored the muscle function decline occurred earlier than muscle mass loss during aging process; older age, adiposity, and malnutrition were implicated with this transition. Few studies have reported the deteriorating effect of obesity on muscle strength (47). To the best of our knowledge, this is the first study that presented the correlations between different obesity indicators and the transition to dynapenia, presarcopenia and sarcopenia. We used AWGS cutoff points to categorize the transition status in a longitudinal study rather than observing changes in single muscle health parameters. By jointly using age, FMR and serum albumin level, we present a cost-efficient and convenient way to predict the risk of dynapenia transition in clinical practice.

Some limitations in this study should be acknowledged. First, the data were retrieved from community-dwelling elderly individuals in Taiwan, and the generalizability of our results to other populations may be limited. Second, applying BIA for body composition estimation is not a standard method. However, adequate hydration and exclusion of severe obesity cases were performed in our study to obtain reliable results. Last, intramuscular or intermuscular fat was difficult to accurately quantify by BIA. Nevertheless, FMR, measured by BIA in this study, reflects fat and muscle distribution and is a better predictor of muscle function decline.

In conclusion, we disclosed that muscle function decline was more prevalent and earlier than muscle mass loss during aging process. Older age, obesity and poor nutrition were risk factors for healthy old people on dynapenia transition. A dose-response effect existed between these three factors and the risk of transition from robust to dynapenia. Older adults with two or more than two of these factors will increase the risk of dynapenia transition than those without.

## Data Availability Statement

The raw data supporting the conclusions of this article will be made available by the authors, without undue reservation.

## Ethics Statement

The studies involving human participants were reviewed and approved by Institution Review Board, Tri-Service General Hospital. The patients/participants provided their written informed consent to participate in this study.

## Author Contributions

T-WK designed the study, analyzed and interpreted the data, and revised the manuscript. Y-PC contributed to manuscript writing. W-HF contributed to perform the statistical analysis and data interpretation. W-LC conceived the study design, participant recruitment, data acquisition, and interpretation. W-SY contributed to study design and data interpretation. T-CP contributed to data acquisition. All authors contributed to the article and approved the submitted version.

## Conflict of Interest

The authors declare that the research was conducted in the absence of any commercial or financial relationships that could be construed as a potential conflict of interest.

## Publisher’s Note

All claims expressed in this article are solely those of the authors and do not necessarily represent those of their affiliated organizations, or those of the publisher, the editors and the reviewers. Any product that may be evaluated in this article, or claim that may be made by its manufacturer, is not guaranteed or endorsed by the publisher.
